# Supplementary material for: Differential diffusion driven far-from-equilibrium shape-shifting of hydrogels
Source: Nat Commun. 2021 Oct 25;12:6155. doi: 10.1038/s41467-021-26464-9 (PMC8546058; doi:10.1038/s41467-021-26464-9)
Supplement: Supplementary file 1 — Supplementary Information [file 41467_2021_26464_MOESM1_ESM.pdf]

Supplementary Information

Differential diffusion driven far-from-equilibrium shape-shifting of hydrogels

Yue Zhang,[1,2] Kangkang Liu,[1] Tao Liu,[3] Chujun Ni,[1] Di Chen,[1] Jiamei Guo,[3] Chang Liu,[1] Jian Zhou,[1] Zheng Jia,[3] Qian Zhao,[1,2*] Pengju Pan,[1] Tao Xie[1,2]

[1]State Key Laboratory of Chemical Engineering, College of Chemical and Biological Engineering, Zhejiang University, Hangzhou 310027, China

[2]ZJU-Hangzhou Global Scientific and Technological Innovation Center, Hangzhou 311215, China

[3]Key Laboratory of Soft Machines and Smart Devices of Zhejiang Province, Center for X-Mechanics, Department of Engineering Mechanics, Zhejiang University, Hangzhou 310027, China

* Correspondence and requests for materials should be addressed to: Q.Z. (email: qianzhao@zju.edu.cn)

This PDF file includes:

Supplementary Figure 1 to 17
Supplementary Table 1.

Other Supplementary Materials for this manuscript include the following:

Movies S1 to S3

Supplementary Figures:

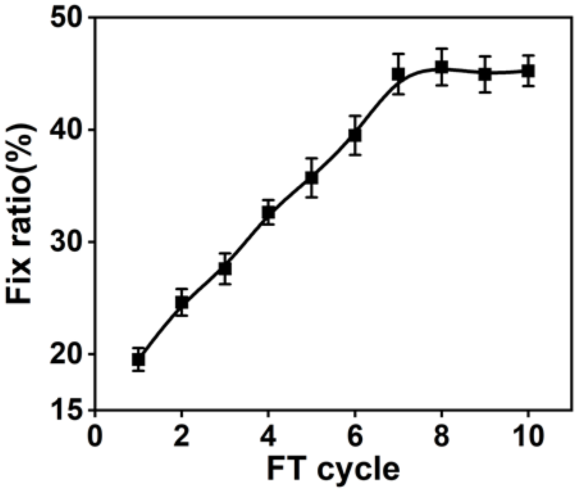

Supplementary Figure 1. Strain fix ratio of the hydrogel upon consecutive freeze-thawing (programming strain: 100%). The error bars were obtained from five parallel experiments.

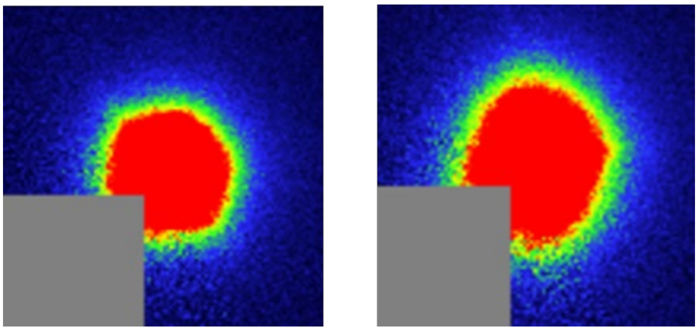

Supplementary Figure 2. 2D SAXS patterns of the isotropic hydrogel (left) and anisotropic hydrogel (right). The isotropic gel was obtained by freezing-thawing 7 times of a swollen hydrogel without external stretching. In contrast, the anisotropic hydrogel is freezing-thawed 7 times with a stretching strain of 100% present in the process.

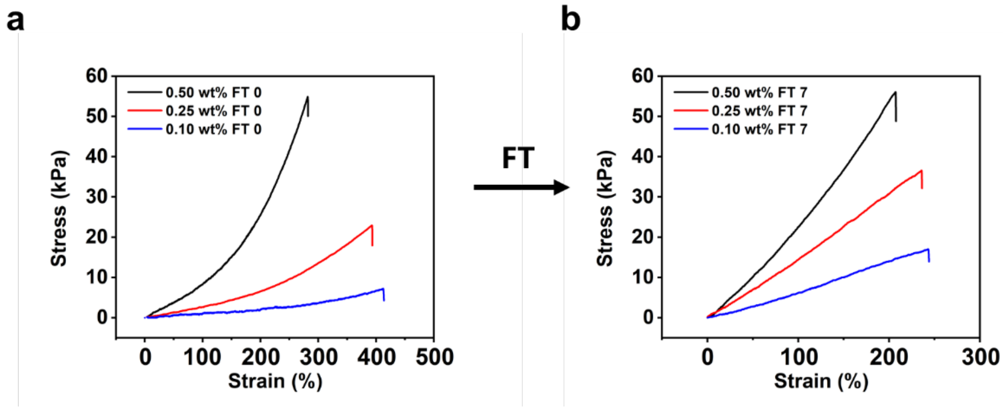

Supplementary Figure 3. Tensile test of the hydrogels prepared from various MBA content (0.10 wt%, 0.25 wt%, and 0.50 wt% of the NIPAM monomer) at a constant concentration of the PVA solution (10 wt%) before (a) and after freeze-thawing (b).

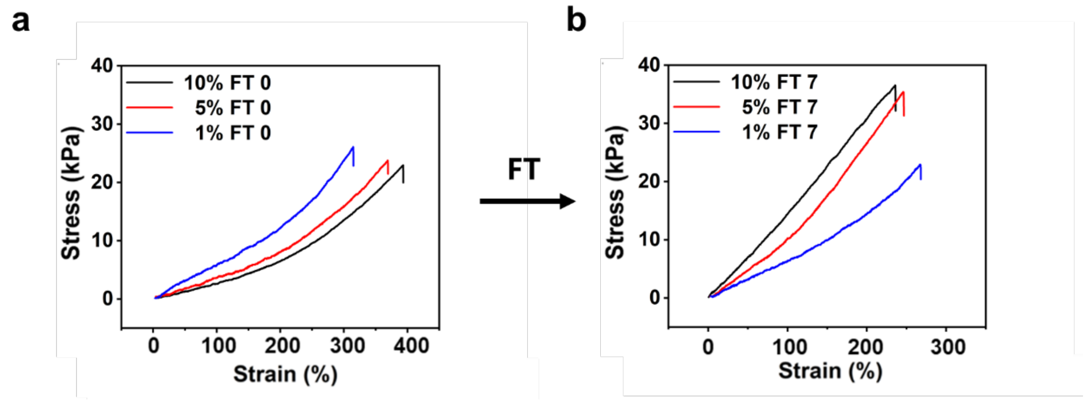

Supplementary Figure 4. Tensile test of the hydrogels prepared from PVA solution with various concentration (1 wt%, 5 wt%, and 10 wt%) at a constant MBA feed (0.25 wt% of the NIPAM monomer) before (a) and after freeze-thawing (b).

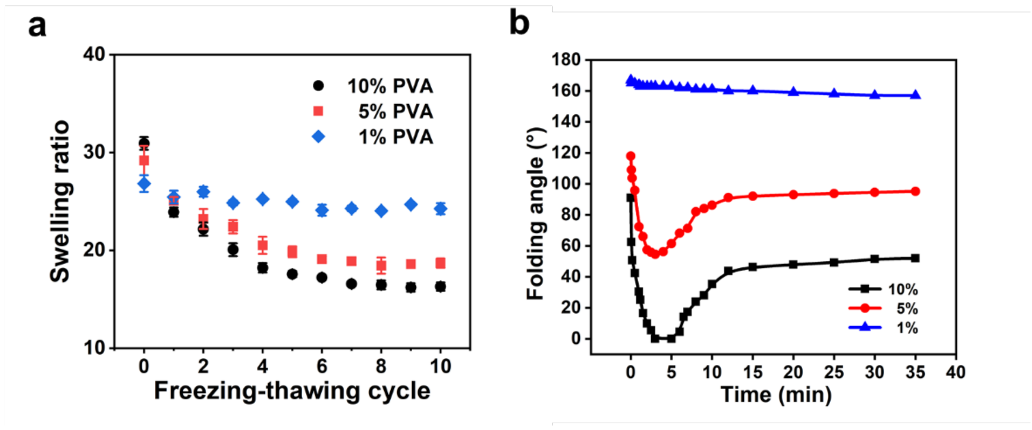

Supplementary Figure 5. Effect of PVA content on swelling ratio and the FFE behavior. (a). Swelling ratios of hydrogels with different PVA content under freeze-thawing cycles; (b). Kinetics of the FFE morphing process upon cooling for hydrogels with different PVA after freeze-thawing for 7 cycles.

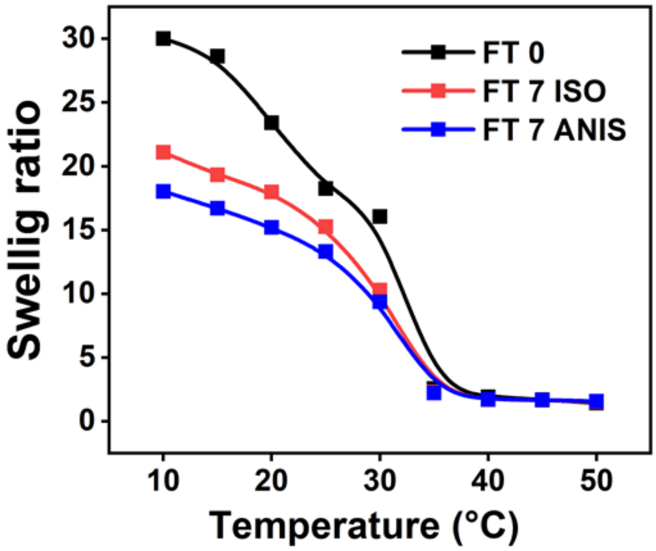

Supplementary Figure 6. Swelling ratios of the hydrogels at various temperature (Black and red lines represent samples with 0 and 7 times freezing-thawing, respectively; blue lines represent an anisotropic sample with 100% programming strain and 7 times freezing-thawing). All three curves show transitions around 32 °C.

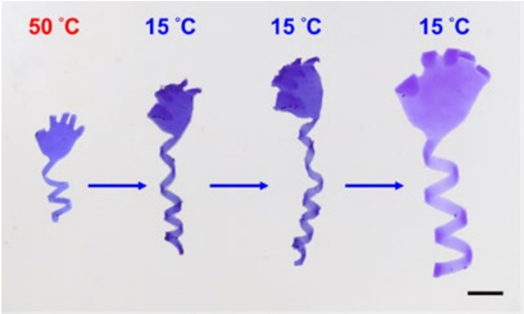

Supplementary Figure 7. The FFE shape-shifting process of a helix-hand hydrogel, in which the helix tail coils and then uncoils while the fingers close and then open. Scale bar: 0.5 cm.

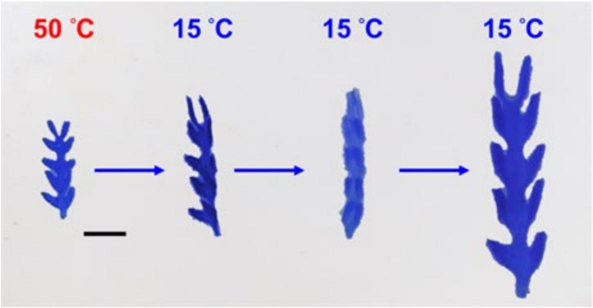

Supplementary Figure 8. The FFE shape-shifting process mimicking the actions of mimosa pudica, in which the leaves close and then open. Scale bar: 0.5 cm.

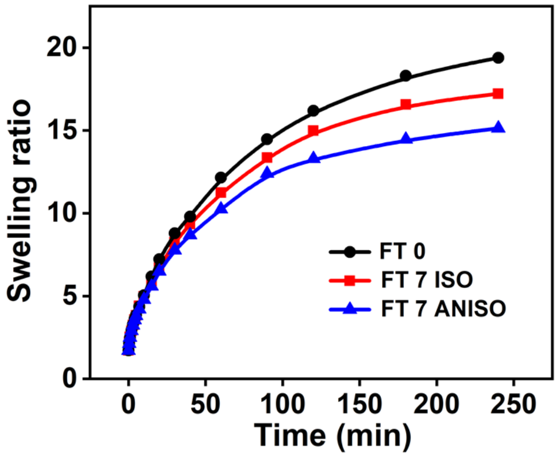

Supplementary Figure 9. Swelling kinetics of as-prepared, anisotropic (programmed strain: 100%) and isotropic gels.

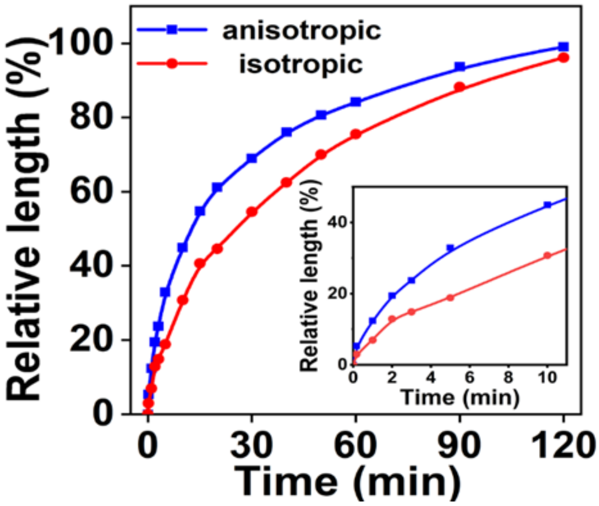

Supplementary Figure 10. Relative length change of anisotropic (programmed strain: 100%) and isotropic gels upon swelling.

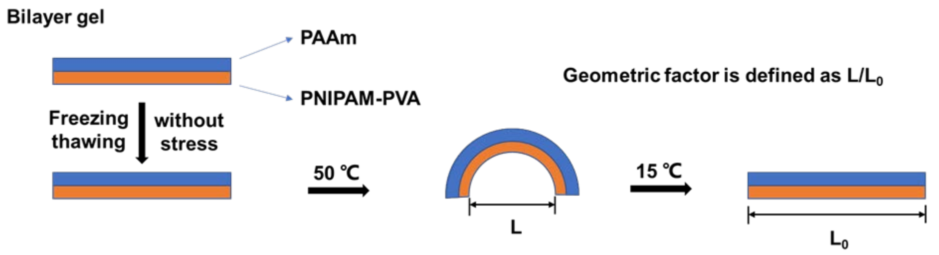

Supplementary Figure 11. Fabrication of a shape-shifting bilayer hydrogel. A thin film of PNIPAM-PVA gel (1 mm) was first fabricated according to the process in the Materials and Methods section. An additional poly(acrylamide) (PAAm) layer (1 mm) was fabricated on top of the PNIPAM-PVA layer by polymerizing acrylamide. The initiator and polymerization conditions were identical to the process for making PNIPAM-PVA.

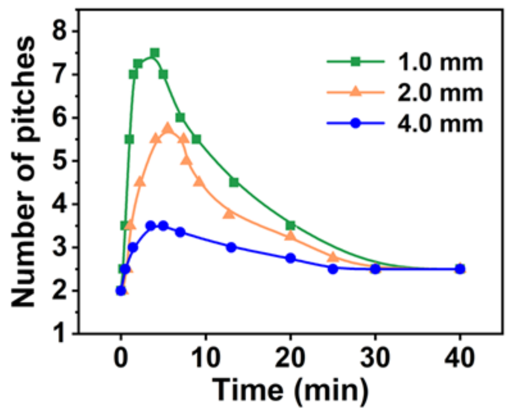

Supplementary Figure 12. The impact of sample width on the FFE behavior of twisted samples. All sample length and thickness were fixed at 20 mm and 0.7 mm, respectively.

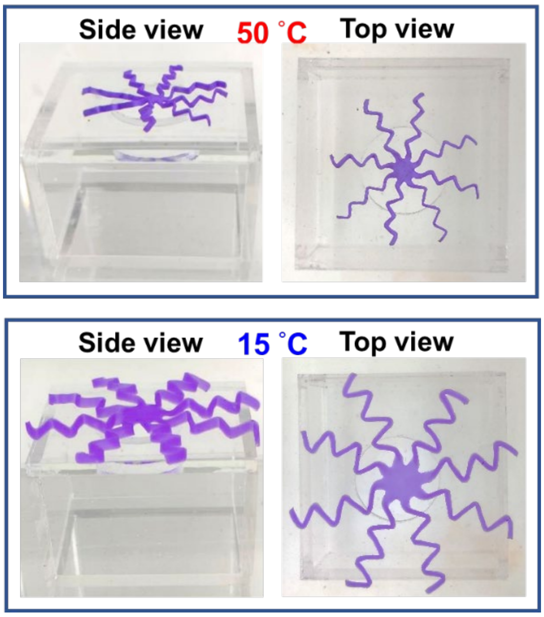

Supplementary Figure 13. Side and top views of the device at the two equilibrium states, showing its much bigger dimension relative to the hole on a box.

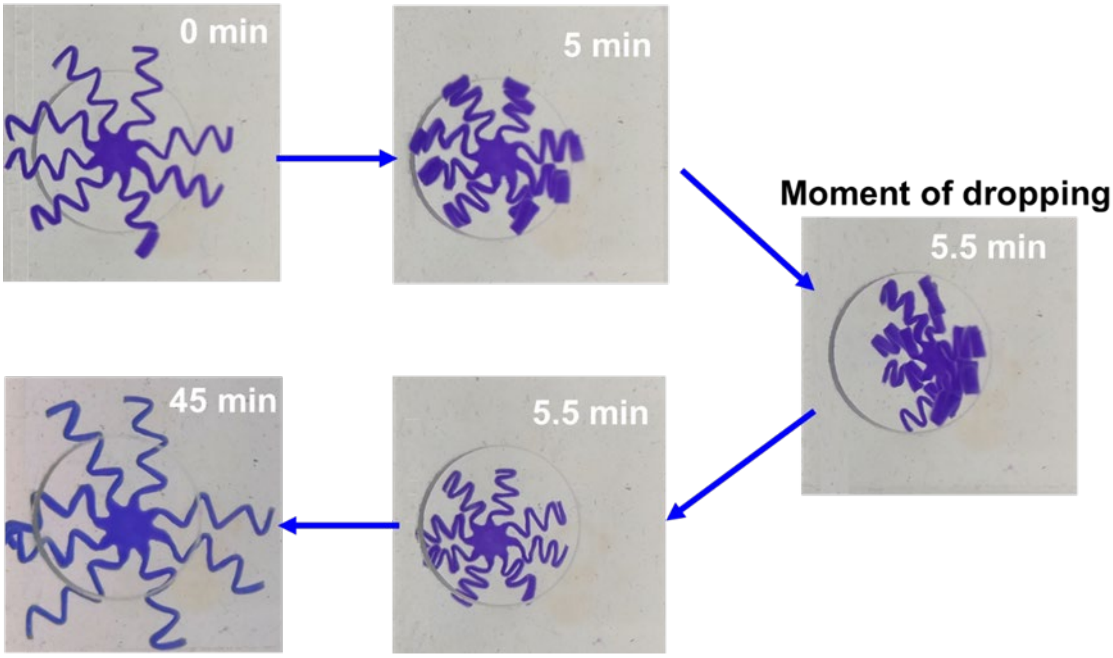

Supplementary Figure 14. Top view of the eight-arm device showing shrinking, dropping, and expanding.

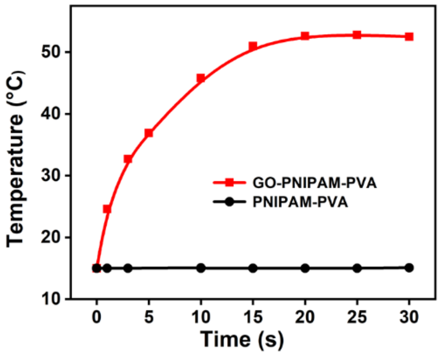

Supplementary Figure 15. Temperature change of hydrogels with and without the photothermal capability. Distance from light to hydrogel: 10 cm. (Light source: 808 nm, 1mW/cm^2)

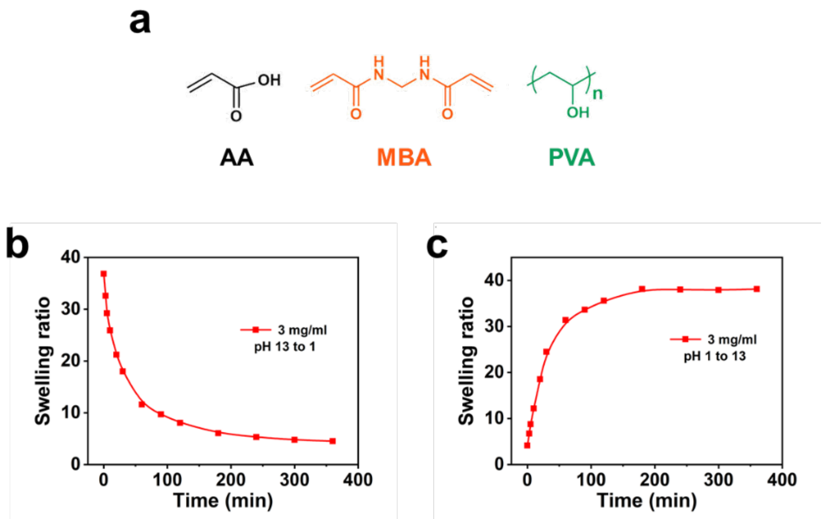

Supplementary Figure 16. Network composition (a) and swelling ratio changes (b, c) in different pH environments of the pH-sensitive FFE shape-morphing hydrogels.

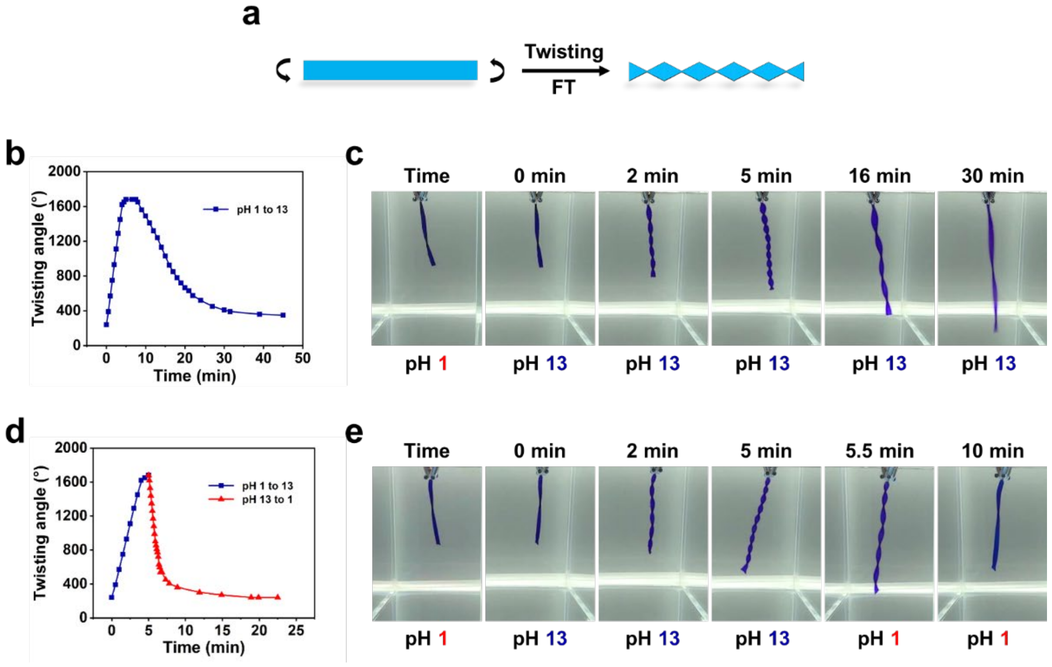

Supplementary Figure 17. (a) Schematic illustration of sample preparation. Quantitative characterization (b) and photographs (c) of the twisting samples from pH=1 to pH=13. Quantitative characterization (d) and photographs (e) of the twisting samples from pH=1 to pH=13 and then pH=1.

Supplementary Table 1. Comparison between mass-diffusion model and thermal-diffusion model

	Mass Diffusion	Thermal Diffusion
Governing equation	Kinetics: $\vec{J} = -D\vec{\nabla}C$<br>Equilibrium: $\frac{\partial C}{\partial t} = \vec{\nabla}(D\vec{\nabla}C)$<br>C is the concentration. D is the diffusion coefficient.	Kinetics: $\vec{q} = -k\vec{\nabla}T$<br>Equilibrium: $\frac{\partial T}{\partial t} = \vec{\nabla}\left(\frac{k}{\rho c}\vec{\nabla}T\right)$<br>k is the thermal conductivity, ρ is the density and c is the specific heat.
Volumetric strain	$\gamma_v \Delta C$<br>γ_v is the diffusion-induced expansion coefficient	$\alpha_v \Delta T$<br>α_v is the thermal expansion coefficient
