# Supplementary material for: Differential diffusion driven far-from-equilibrium shape-shifting of hydrogels
Source: Nat Commun. 2021 Oct 25;12:6155. doi: 10.1038/s41467-021-26464-9 (PMC8546058; doi:10.1038/s41467-021-26464-9)
Supplement: Supplementary file 3 — Description of Additional Supplementary Files [file 41467_2021_26464_MOESM3_ESM.pdf]

Description of Additional Supplementary Files

File Name: Supplementary Movie 1
Description: FFE behavior of zig-zag samples in 15 °C water.

File Name: Supplementary Movie 2
Description: Twisting and untwisting morphing of the twisted sample in 15 °C water. In order to enhance the visual effect, we fixed a small fan blade at the bottom of the spline.

File Name: Supplementary Movie 3
Description: Video showing the eight-arm device shrinking, dropping and expanding in 15 °C water.
